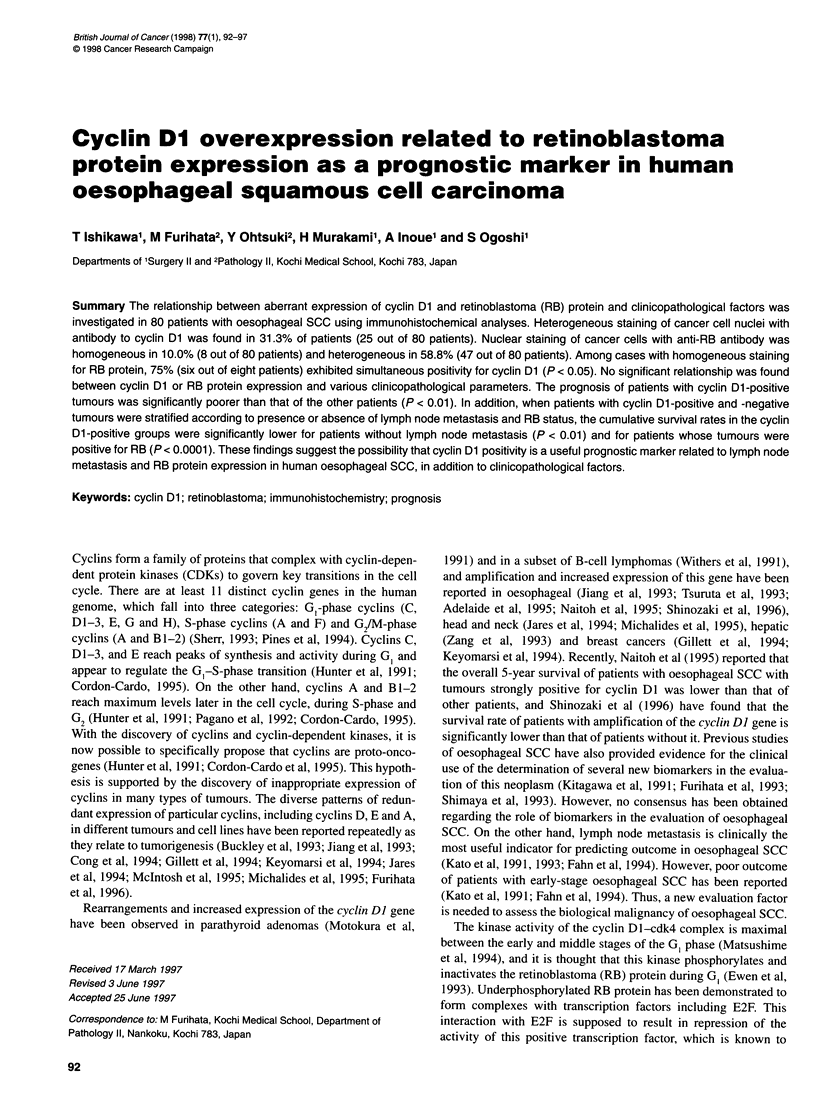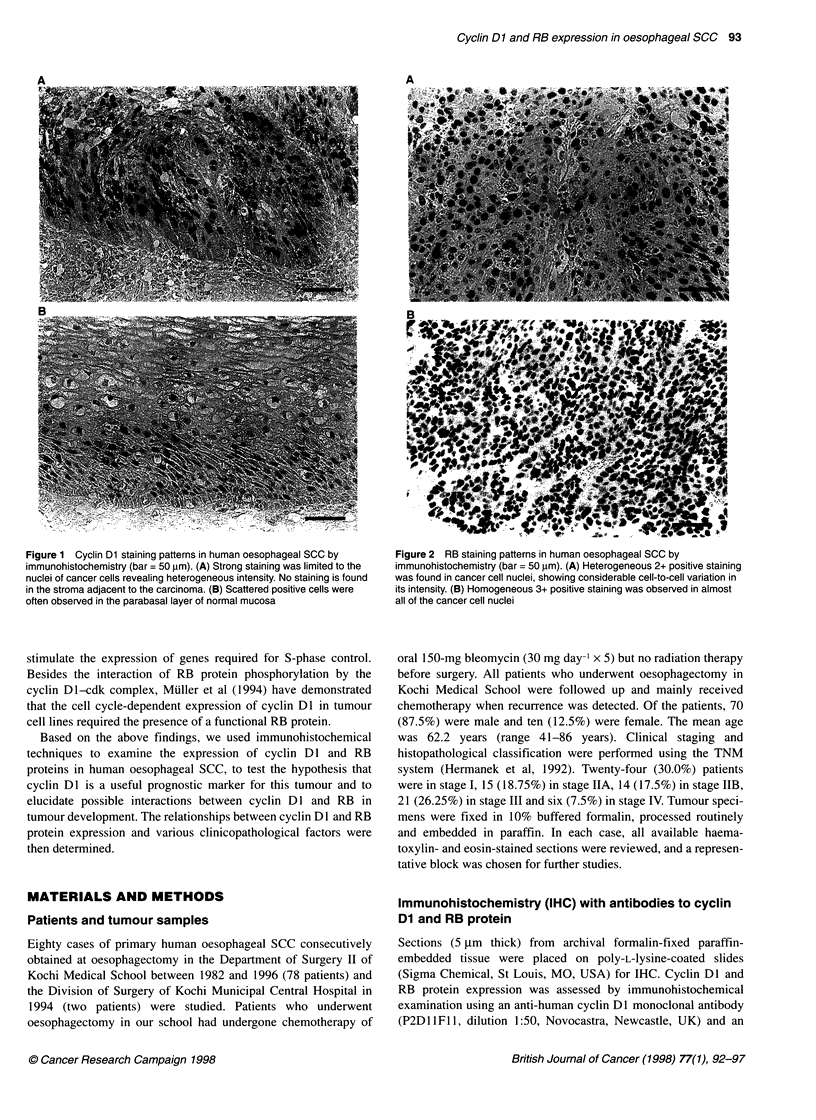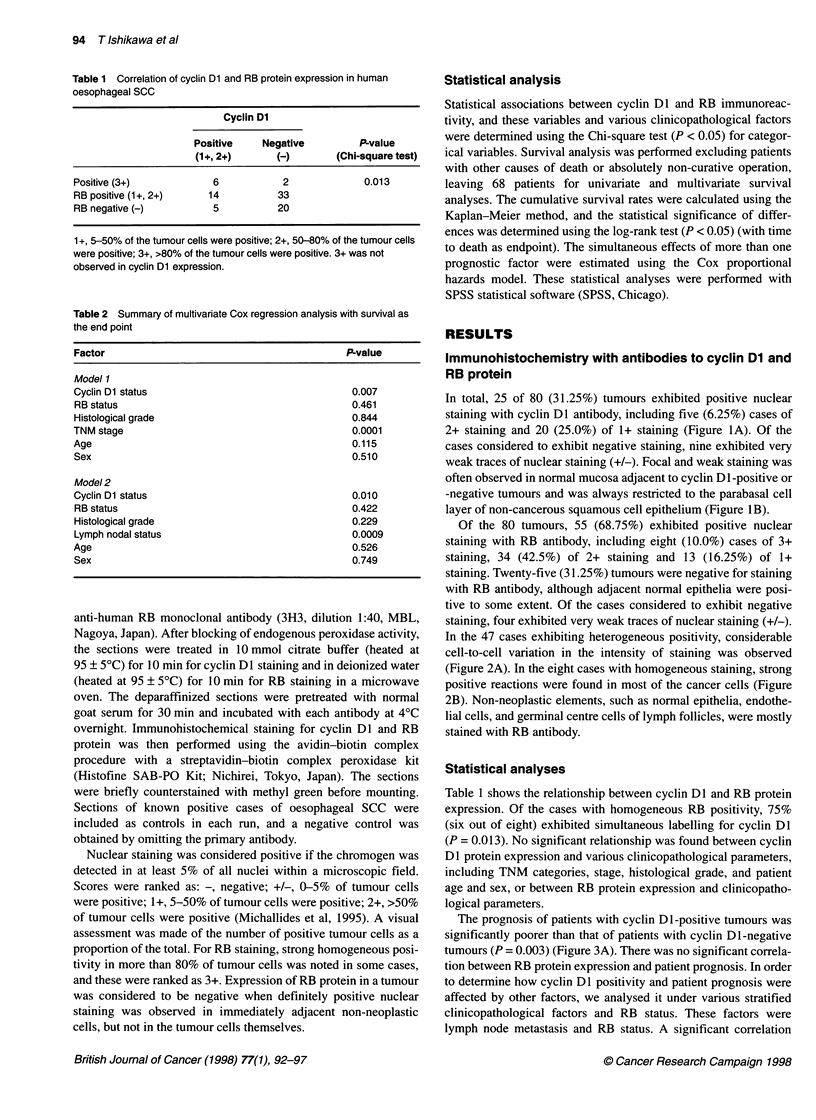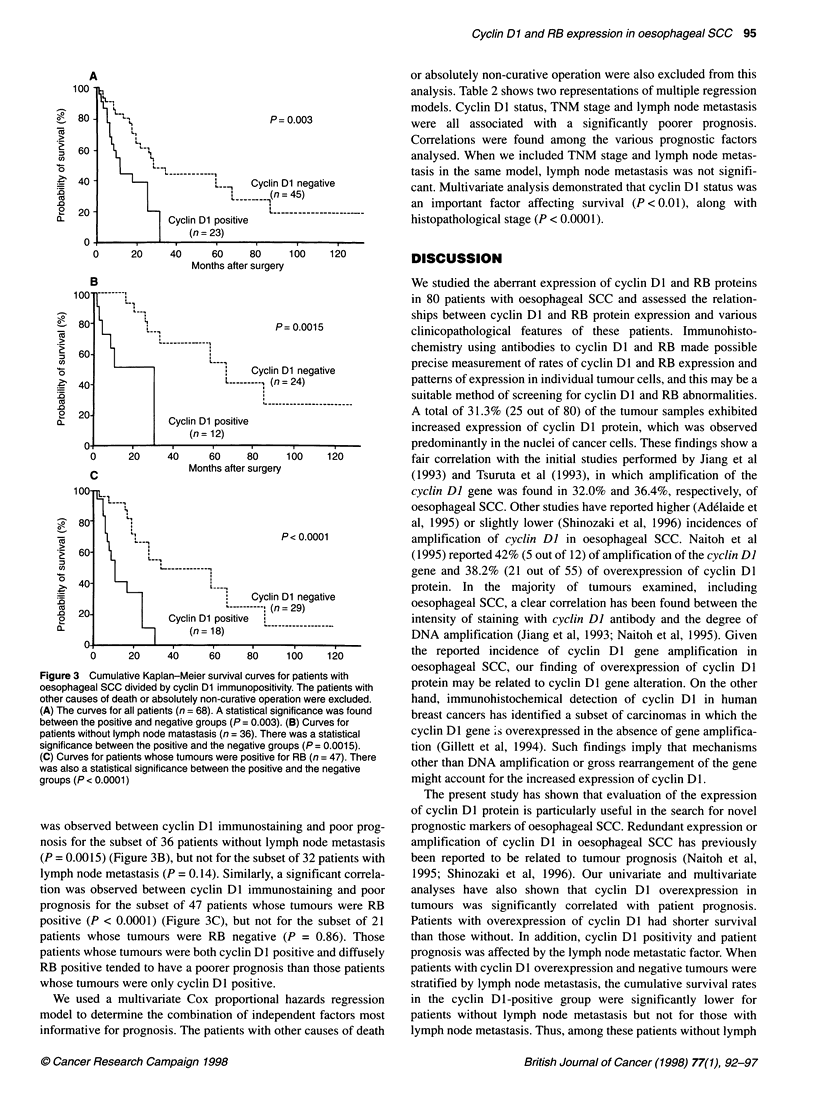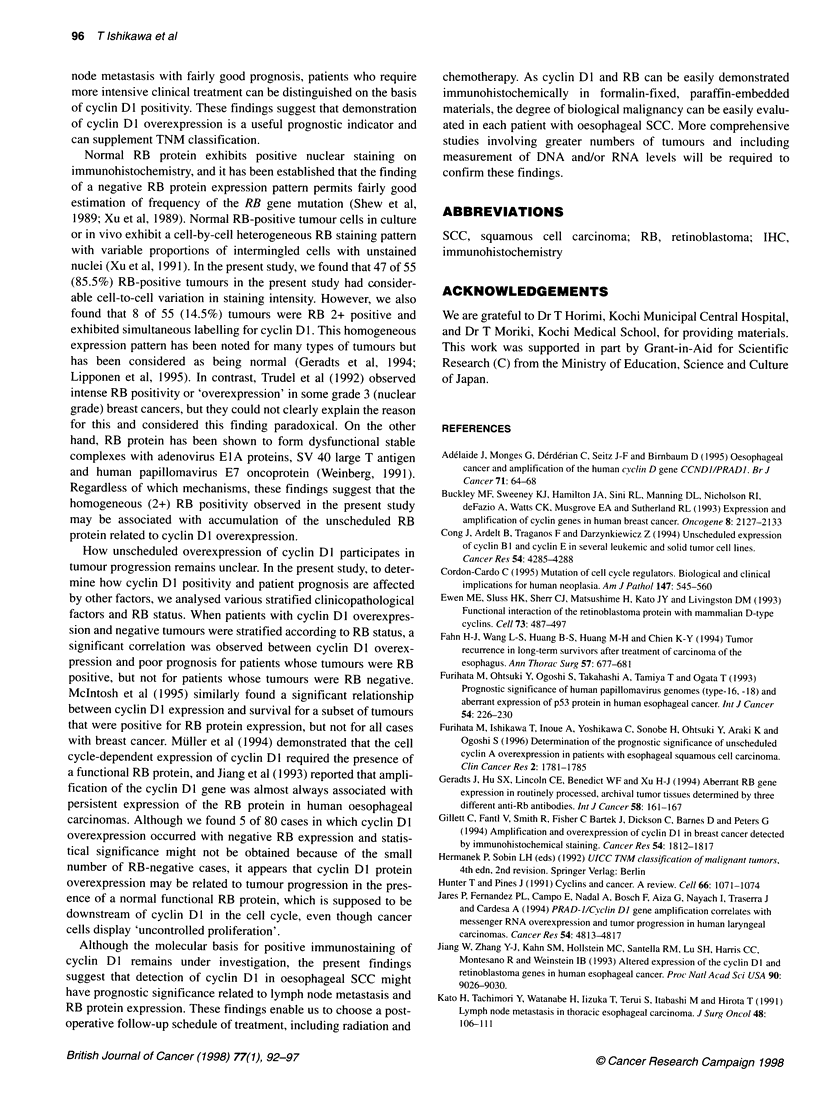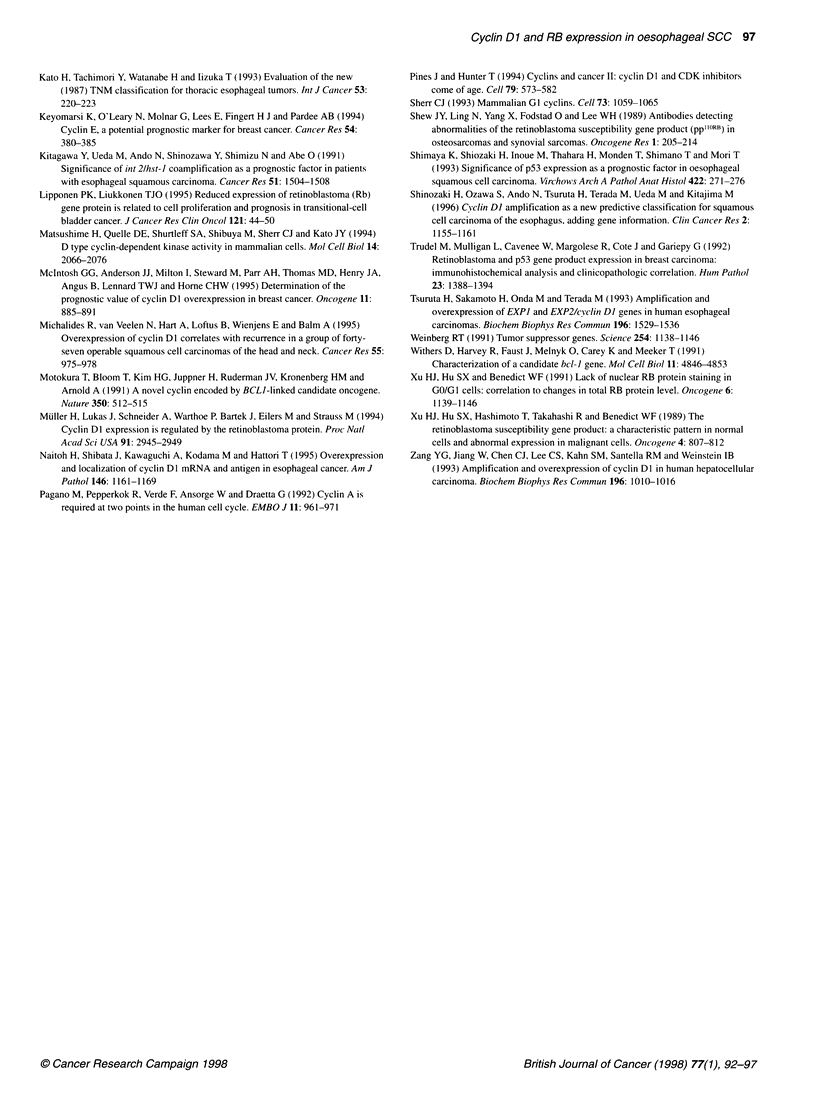# Cyclin D1 overexpression related to retinoblastoma protein expression as a prognostic marker in human oesophageal squamous cell carcinoma.

**DOI:** 10.1038/bjc.1998.14

**Published:** 1998

**Authors:** T. Ishikawa, M. Furihata, Y. Ohtsuki, H. Murakami, A. Inoue, S. Ogoshi

**Affiliations:** Department of Surgery II, Kochi Medical School, Japan.

## Abstract

**Images:**


British Journal of Cancer (1998) 77(1), 92–97
© 1998 Cancer Research Campaign

### Cyclin D1 overexpression related to retinoblastoma protein expression as a prognostic marker in human oesophageal squamous cell carcinoma

**T Ishikawa[1], M Furihata[2], Y Ohtsuki[2], H Murakami[1], A Inoue[1] and S Ogoshi[1]**

Departments of [1]Surgery II and [2]Pathology II, Kochi Medical School, Kochi 783, Japan

**Summary** The relationship between aberrant expression of cyclin D1 and retinoblastoma (RB) protein and clinicopathological factors was investigated in 80 patients with oesophageal SCC using immunohistochemical analyses. Heterogeneous staining of cancer cell nuclei with antibody to cyclin D1 was found in 31.3% of patients (25 out of 80 patients). Nuclear staining of cancer cells with anti-RB antibody was homogeneous in 10.0% (8 out of 80 patients) and heterogeneous in 58.8% (47 out of 80 patients). Among cases with homogeneous staining for RB protein, 75% (six out of eight patients) exhibited simultaneous positivity for cyclin D1 ($P < 0.05$). No significant relationship was found between cyclin D1 or RB protein expression and various clinicopathological parameters. The prognosis of patients with cyclin D1-positive tumours was significantly poorer than that of the other patients ($P < 0.01$). In addition, when patients with cyclin D1-positive and -negative tumours were stratified according to presence or absence of lymph node metastasis and RB status, the cumulative survival rates in the cyclin D1-positive groups were significantly lower for patients without lymph node metastasis ($P < 0.01$) and for patients whose tumours were positive for RB ($P < 0.0001$). These findings suggest the possibility that cyclin D1 positivity is a useful prognostic marker related to lymph node metastasis and RB protein expression in human oesophageal SCC, in addition to clinicopathological factors.

**Keywords:** cyclin D1; retinoblastoma; immunohistochemistry; prognosis

Cyclins form a family of proteins that complex with cyclin-dependent protein kinases (CDKs) to govern key transitions in the cell cycle. There are at least 11 distinct cyclin genes in the human genome, which fall into three categories: $G_1$-phase cyclins (C, D1–3, E, G and H), S-phase cyclins (A and F) and $G_2$/M-phase cyclins (A and B1–2) (Sherr, 1993; Pines et al, 1994). Cyclins C, D1–3, and E reach peaks of synthesis and activity during $G_1$ and appear to regulate the $G_1$–S-phase transition (Hunter et al, 1991; Cordon-Cardo, 1995). On the other hand, cyclins A and B1–2 reach maximum levels later in the cell cycle, during S-phase and $G_2$ (Hunter et al, 1991; Pagano et al, 1992; Cordon-Cardo, 1995). With the discovery of cyclins and cyclin-dependent kinases, it is now possible to specifically propose that cyclins are proto-oncogenes (Hunter et al, 1991; Cordon-Cardo et al, 1995). This hypothesis is supported by the discovery of inappropriate expression of cyclins in many types of tumours. The diverse patterns of redundant expression of particular cyclins, including cyclins D, E and A, in different tumours and cell lines have been reported repeatedly as they relate to tumorigenesis (Buckley et al, 1993; Jiang et al, 1993; Cong et al, 1994; Gillett et al, 1994; Keyomarsi et al, 1994; Jares et al, 1994; McIntosh et al, 1995; Michalides et al, 1995; Furihata et al, 1996).

Rearrangements and increased expression of the *cyclin D1* gene have been observed in parathyroid adenomas (Motokura et al, 1991) and in a subset of B-cell lymphomas (Withers et al, 1991), and amplification and increased expression of this gene have been reported in oesophageal (Jiang et al, 1993; Tsuruta et al, 1993; Adelaide et al, 1995; Naitoh et al, 1995; Shinozaki et al, 1996), head and neck (Jares et al, 1994; Michalides et al, 1995), hepatic (Zang et al, 1993) and breast cancers (Gillett et al, 1994; Keyomarsi et al, 1994). Recently, Naitoh et al (1995) reported that the overall 5-year survival of patients with oesophageal SCC with tumours strongly positive for cyclin D1 was lower than that of other patients, and Shinozaki et al (1996) have found that the survival rate of patients with amplification of the *cyclin D1* gene is significantly lower than that of patients without it. Previous studies of oesophageal SCC have also provided evidence for the clinical use of the determination of several new biomarkers in the evaluation of this neoplasm (Kitagawa et al, 1991; Furihata et al, 1993; Shimaya et al, 1993). However, no consensus has been obtained regarding the role of biomarkers in the evaluation of oesophageal SCC. On the other hand, lymph node metastasis is clinically the most useful indicator for predicting outcome in oesophageal SCC (Kato et al, 1991, 1993; Fahn et al, 1994). However, poor outcome of patients with early-stage oesophageal SCC has been reported (Kato et al, 1991; Fahn et al, 1994). Thus, a new evaluation factor is needed to assess the biological malignancy of oesophageal SCC.

The kinase activity of the cyclin D1–cdk4 complex is maximal between the early and middle stages of the $G_1$ phase (Matsushime et al, 1994), and it is thought that this kinase phosphorylates and inactivates the retinoblastoma (RB) protein during $G_1$ (Ewen et al, 1993). Underphosphorylated RB protein has been demonstrated to form complexes with transcription factors including E2F. This interaction with E2F is supposed to result in repression of the activity of this positive transcription factor, which is known to

*Received 17 March 1997*
*Revised 3 June 1997*
*Accepted 25 June 1997*

*Correspondence to:* M Furihata, Kochi Medical School, Department of Pathology II, Nankoku, Kochi 783, Japan

**Figure 1** Cyclin D1 staining patterns in human oesophageal SCC by immunohistochemistry (bar = 50 µm). (**A**) Strong staining was limited to the nuclei of cancer cells revealing heterogeneous intensity. No staining is found in the stroma adjacent to the carcinoma. (**B**) Scattered positive cells were often observed in the parabasal layer of normal mucosa

**Figure 2** RB staining patterns in human oesophageal SCC by immunohistochemistry (bar = 50 µm). (**A**) Heterogeneous 2+ positive staining was found in cancer cell nuclei, showing considerable cell-to-cell variation in its intensity. (**B**) Homogeneous 3+ positive staining was observed in almost all of the cancer cell nuclei stimulate the expression of genes required for S-phase control. Besides the interaction of RB protein phosphorylation by the cyclin D1–cdk complex, Müller et al (1994) have demonstrated that the cell cycle-dependent expression of cyclin D1 in tumour cell lines required the presence of a functional RB protein.

Based on the above findings, we used immunohistochemical techniques to examine the expression of cyclin D1 and RB proteins in human oesophageal SCC, to test the hypothesis that cyclin D1 is a useful prognostic marker for this tumour and to elucidate possible interactions between cyclin D1 and RB in tumour development. The relationships between cyclin D1 and RB protein expression and various clinicopathological factors were then determined.

### MATERIALS AND METHODS

### Patients and tumour samples

Eighty cases of primary human oesophageal SCC consecutively obtained at oesophagectomy in the Department of Surgery II of Kochi Medical School between 1982 and 1996 (78 patients) and the Division of Surgery of Kochi Municipal Central Hospital in 1994 (two patients) were studied. Patients who underwent oesophagectomy in our school had undergone chemotherapy of oral 150-mg bleomycin (30 mg $day^{-1} \times 5$) but no radiation therapy before surgery. All patients who underwent oesophagectomy in Kochi Medical School were followed up and mainly received chemotherapy when recurrence was detected. Of the patients, 70 (87.5%) were male and ten (12.5%) were female. The mean age was 62.2 years (range 41–86 years). Clinical staging and histopathological classification were performed using the TNM system (Hermanek et al, 1992). Twenty-four (30.0%) patients were in stage I, 15 (18.75%) in stage IIA, 14 (17.5%) in stage IIB, 21 (26.25%) in stage III and six (7.5%) in stage IV. Tumour specimens were fixed in 10% buffered formalin, processed routinely and embedded in paraffin. In each case, all available haematoxylin- and eosin-stained sections were reviewed, and a representative block was chosen for further studies.

### Immunohistochemistry (IHC) with antibodies to cyclin D1 and RB protein

Sections (5 µm thick) from archival formalin-fixed paraffin-embedded tissue were placed on poly-L-lysine-coated slides (Sigma Chemical, St Louis, MO, USA) for IHC. Cyclin D1 and RB protein expression was assessed by immunohistochemical examination using an anti-human cyclin D1 monoclonal antibody (P2D11F11, dilution 1:50, Novocastra, Newcastle, UK) and an

© Cancer Research Campaign 1998

**Table 1** Correlation of cyclin D1 and RB protein expression in human oesophageal SCC

| | Cyclin D1 | | |
|---|---|---|---|
| | Positive (1+, 2+) | Negative (–) | *P*-value (Chi-square test) |
| Positive (3+) | 6 | 2 | 0.013 |
| RB positive (1+, 2+) | 14 | 33 | |
| RB negative (–) | 5 | 20 | |

1+, 5–50% of the tumour cells were positive; 2+, 50–80% of the tumour cells were positive; 3+, >80% of the tumour cells were positive. 3+ was not observed in cyclin D1 expression.

**Table 2** Summary of multivariate Cox regression analysis with survival as the end point

| Factor | *P*-value |
|---|---|
| *Model 1* | |
| Cyclin D1 status | 0.007 |
| RB status | 0.461 |
| Histological grade | 0.844 |
| TNM stage | 0.0001 |
| Age | 0.115 |
| Sex | 0.510 |
| *Model 2* | |
| Cyclin D1 status | 0.010 |
| RB status | 0.422 |
| Histological grade | 0.229 |
| Lymph nodal status | 0.0009 |
| Age | 0.526 |
| Sex | 0.749 |

anti-human RB monoclonal antibody (3H3, dilution 1:40, MBL, Nagoya, Japan). After blocking of endogenous peroxidase activity, the sections were treated in 10 mmol citrate buffer (heated at 95 ± 5°C) for 10 min for cyclin D1 staining and in deionized water (heated at 95 ± 5°C) for 10 min for RB staining in a microwave oven. The deparaffinized sections were pretreated with normal goat serum for 30 min and incubated with each antibody at 4°C overnight. Immunohistochemical staining for cyclin D1 and RB protein was then performed using the avidin–biotin complex procedure with a streptavidin-biotin complex peroxidase kit (Histofine SAB-PO Kit; Nichirei, Tokyo, Japan). The sections were briefly counterstained with methyl green before mounting. Sections of known positive cases of oesophageal SCC were included as controls in each run, and a negative control was obtained by omitting the primary antibody.

Nuclear staining was considered positive if the chromogen was detected in at least 5% of all nuclei within a microscopic field. Scores were ranked as: –, negative; +/–, 0–5% of tumour cells were positive; 1+, 5–50% of tumour cells were positive; 2+, >50% of tumour cells were positive (Michallides et al, 1995). A visual assessment was made of the number of positive tumour cells as a proportion of the total. For RB staining, strong homogeneous positivity in more than 80% of tumour cells was noted in some cases, and these were ranked as 3+. Expression of RB protein in a tumour was considered to be negative when definitely positive nuclear staining was observed in immediately adjacent non-neoplastic cells, but not in the tumour cells themselves.

### Statistical analysis

Statistical associations between cyclin D1 and RB immunoreactivity, and these variables and various clinicopathological factors were determined using the Chi-square test ($P < 0.05$) for categorical variables. Survival analysis was performed excluding patients with other causes of death or absolutely non-curative operation, leaving 68 patients for univariate and multivariate survival analyses. The cumulative survival rates were calculated using the Kaplan–Meier method, and the statistical significance of differences was determined using the log-rank test ($P < 0.05$) (with time to death as endpoint). The simultaneous effects of more than one prognostic factor were estimated using the Cox proportional hazards model. These statistical analyses were performed with SPSS statistical software (SPSS, Chicago).

### RESULTS

### Immunohistochemistry with antibodies to cyclin D1 and RB protein

In total, 25 of 80 (31.25%) tumours exhibited positive nuclear staining with cyclin D1 antibody, including five (6.25%) cases of 2+ staining and 20 (25.0%) of 1+ staining (Figure 1A). Of the cases considered to exhibit negative staining, nine exhibited very weak traces of nuclear staining (+/–). Focal and weak staining was often observed in normal mucosa adjacent to cyclin D1-positive or -negative tumours and was always restricted to the parabasal cell layer of non-cancerous squamous cell epithelium (Figure 1B).

Of the 80 tumours, 55 (68.75%) exhibited positive nuclear staining with RB antibody, including eight (10.0%) cases of 3+ staining, 34 (42.5%) of 2+ staining and 13 (16.25%) of 1+ staining. Twenty-five (31.25%) tumours were negative for staining with RB antibody, although adjacent normal epithelia were positive to some extent. Of the cases considered to exhibit negative staining, four exhibited very weak traces of nuclear staining (+/–). In the 47 cases exhibiting heterogeneous positivity, considerable cell-to-cell variation in the intensity of staining was observed (Figure 2A). In the eight cases with homogeneous staining, strong positive reactions were found in most of the cancer cells (Figure 2B). Non-neoplastic elements, such as normal epithelia, endothelial cells, and germinal centre cells of lymph follicles, were mostly stained with RB antibody.

### Statistical analyses

Table 1 shows the relationship between cyclin D1 and RB protein expression. Of the cases with homogeneous RB positivity, 75% (six out of eight) exhibited simultaneous labelling for cyclin D1 ($P = 0.013$). No significant relationship was found between cyclin D1 protein expression and various clinicopathological parameters, including TNM categories, stage, histological grade, and patient age and sex, or between RB protein expression and clinicopathological parameters.

The prognosis of patients with cyclin D1-positive tumours was significantly poorer than that of patients with cyclin D1-negative tumours ($P = 0.003$) (Figure 3A). There was no significant correlation between RB protein expression and patient prognosis. In order to determine how cyclin D1 positivity and patient prognosis were affected by other factors, we analysed it under various stratified clinicopathological factors and RB status. These factors were lymph node metastasis and RB status. A significant correlation

© Cancer Research Campaign 1998

**Figure 3** Cumulative Kaplan–Meier survival curves for patients with oesophageal SCC divided by cyclin D1 immunopositivity. The patients with other causes of death or absolutely non-curative operation were excluded. (**A**) The curves for all patients ($n$ = 68). A statistical significance was found between the positive and negative groups ($P$ = 0.003). (**B**) Curves for patients without lymph node matastasis ($n$ = 36). There was a statistical significance between the positive and the negative groups ($P$ = 0.0015). (**C**) Curves for patients whose tumours were positive for RB ($n$ = 47). There was also a statistical significance between the positive and the negative groups ($P$ < 0.0001)

was observed between cyclin D1 immunostaining and poor prognosis for the subset of 36 patients without lymph node metastasis ($P$ = 0.0015) (Figure 3B), but not for the subset of 32 patients with lymph node metastasis ($P$ = 0.14). Similarly, a significant correlation was observed between cyclin D1 immunostaining and poor prognosis for the subset of 47 patients whose tumours were RB positive ($P$ < 0.0001) (Figure 3C), but not for the subset of 21 patients whose tumours were RB negative ($P$ = 0.86). Those patients whose tumours were both cyclin D1 positive and diffusely RB positive tended to have a poorer prognosis than those patients whose tumours were only cyclin D1 positive.

We used a multivariate Cox proportional hazards regression model to determine the combination of independent factors most informative for prognosis. The patients with other causes of death or absolutely non-curative operation were also excluded from this analysis. Table 2 shows two representations of multiple regression models. Cyclin D1 status, TNM stage and lymph node metastasis were all associated with a significantly poorer prognosis. Correlations were found among the various prognostic factors analysed. When we included TNM stage and lymph node metastasis in the same model, lymph node metastasis was not significant. Multivariate analysis demonstrated that cyclin D1 status was an important factor affecting survival ($P$ < 0.01), along with histopathological stage ($P$ < 0.0001).

### DISCUSSION

We studied the aberrant expression of cyclin D1 and RB proteins in 80 patients with oesophageal SCC and assessed the relationships between cyclin D1 and RB protein expression and various clinicopathological features of these patients. Immunohistochemistry using antibodies to cyclin D1 and RB made possible precise measurement of rates of cyclin D1 and RB expression and patterns of expression in individual tumour cells, and this may be a suitable method of screening for cyclin D1 and RB abnormalities. A total of 31.3% (25 out of 80) of the tumour samples exhibited increased expression of cyclin D1 protein, which was observed predominantly in the nuclei of cancer cells. These findings show a fair correlation with the initial studies performed by Jiang et al (1993) and Tsuruta et al (1993), in which amplification of the *cyclin D1* gene was found in 32.0% and 36.4%, respectively, of oesophageal SCC. Other studies have reported higher (Adélaide et al, 1995) or slightly lower (Shinozaki et al, 1996) incidences of amplification of *cyclin D1* in oesophageal SCC. Naitoh et al (1995) reported 42% (5 out of 12) of amplification of the *cyclin D1* gene and 38.2% (21 out of 55) of overexpression of cyclin D1 protein. In the majority of tumours examined, including oesophageal SCC, a clear correlation has been found between the intensity of staining with *cyclin D1* antibody and the degree of DNA amplification (Jiang et al, 1993; Naitoh et al, 1995). Given the reported incidence of cyclin D1 gene amplification in oesophageal SCC, our finding of overexpression of cyclin D1 protein may be related to cyclin D1 gene alteration. On the other hand, immunohistochemical detection of cyclin D1 in human breast cancers has identified a subset of carcinomas in which the cyclin D1 gene is overexpressed in the absence of gene amplification (Gillett et al, 1994). Such findings imply that mechanisms other than DNA amplification or gross rearrangement of the gene might account for the increased expression of cyclin D1.

The present study has shown that evaluation of the expression of cyclin D1 protein is particularly useful in the search for novel prognostic markers of oesophageal SCC. Redundant expression or amplification of cyclin D1 in oesophageal SCC has previously been reported to be related to tumour prognosis (Naitoh et al, 1995; Shinozaki et al, 1996). Our univariate and multivariate analyses have also shown that cyclin D1 overexpression in tumours was significantly correlated with patient prognosis. Patients with overexpression of cyclin D1 had shorter survival than those without. In addition, cyclin D1 positivity and patient prognosis was affected by the lymph node metastatic factor. When patients with cyclin D1 overexpression and negative tumours were stratified by lymph node metastasis, the cumulative survival rates in the cyclin D1-positive group were significantly lower for patients without lymph node metastasis but not for those with lymph node metastasis. Thus, among these patients without lymph

© Cancer Research Campaign 1998 

node metastasis with fairly good prognosis, patients who require more intensive clinical treatment can be distinguished on the basis of cyclin D1 positivity. These findings suggest that demonstration of cyclin D1 overexpression is a useful prognostic indicator and can supplement TNM classification.

Normal RB protein exhibits positive nuclear staining on immunohistochemistry, and it has been established that the finding of a negative RB protein expression pattern permits fairly good estimation of frequency of the *RB* gene mutation (Shew et al, 1989; Xu et al, 1989). Normal RB-positive tumour cells in culture or in vivo exhibit a cell-by-cell heterogeneous RB staining pattern with variable proportions of intermingled cells with unstained nuclei (Xu et al, 1991). In the present study, we found that 47 of 55 (85.5%) RB-positive tumours in the present study had considerable cell-to-cell variation in staining intensity. However, we also found that 8 of 55 (14.5%) tumours were RB 2+ positive and exhibited simultaneous labelling for cyclin D1. This homogeneous expression pattern has been noted for many types of tumours but has been considered as being normal (Geradts et al, 1994; Lipponen et al, 1995). In contrast, Trudel et al (1992) observed intense RB positivity or 'overexpression' in some grade 3 (nuclear grade) breast cancers, but they could not clearly explain the reason for this and considered this finding paradoxical. On the other hand, RB protein has been shown to form dysfunctional stable complexes with adenovirus E1A proteins, SV 40 large T antigen and human papillomavirus E7 oncoprotein (Weinberg, 1991). Regardless of which mechanisms, these findings suggest that the homogeneous (2+) RB positivity observed in the present study may be associated with accumulation of the unscheduled RB protein related to cyclin D1 overexpression.

How unscheduled overexpression of cyclin D1 participates in tumour progression remains unclear. In the present study, to determine how cyclin D1 positivity and patient prognosis are affected by other factors, we analysed various stratified clinicopathological factors and RB status. When patients with cyclin D1 overexpression and negative tumours were stratified according to RB status, a significant correlation was observed between cyclin D1 overexpression and poor prognosis for patients whose tumours were RB positive, but not for patients whose tumours were RB negative. McIntosh et al (1995) similarly found a significant relationship between cyclin D1 expression and survival for a subset of tumours that were positive for RB protein expression, but not for all cases with breast cancer. Müller et al (1994) demonstrated that the cell cycle-dependent expression of cyclin D1 required the presence of a functional RB protein, and Jiang et al (1993) reported that amplification of the cyclin D1 gene was almost always associated with persistent expression of the RB protein in human oesophageal carcinomas. Although we found 5 of 80 cases in which cyclin D1 overexpression occurred with negative RB expression and statistical significance might not be obtained because of the small number of RB-negative cases, it appears that cyclin D1 protein overexpression may be related to tumour progression in the presence of a normal functional RB protein, which is supposed to be downstream of cyclin D1 in the cell cycle, even though cancer cells display 'uncontrolled proliferation'.

Although the molecular basis for positive immunostaining of cyclin D1 remains under investigation, the present findings suggest that detection of cyclin D1 in oesophageal SCC might have prognostic significance related to lymph node metastasis and RB protein expression. These findings enable us to choose a postoperative follow-up schedule of treatment, including radiation and chemotherapy. As cyclin D1 and RB can be easily demonstrated immunohistochemically in formalin-fixed, paraffin-embedded materials, the degree of biological malignancy can be easily evaluated in each patient with oesophageal SCC. More comprehensive studies involving greater numbers of tumours and including measurement of DNA and/or RNA levels will be required to confirm these findings.

### ABBREVIATIONS

SCC, squamous cell carcinoma; RB, retinoblastoma; IHC, immunohistochemistry

### ACKNOWLEDGEMENTS

We are grateful to Dr T Horimi, Kochi Municipal Central Hospital, and Dr T Moriki, Kochi Medical School, for providing materials. This work was supported in part by Grant-in-Aid for Scientific Research (C) from the Ministry of Education, Science and Culture of Japan.

### REFERENCES

Adélaide J, Monges G, Dérdérian C, Seitz J-F and Birnbaum D (1995) Oesophageal cancer and amplification of the human *cyclin D* gene *CCND1/PRAD1*. *Br J Cancer* **71**: 64–68

Buckley MF, Sweeney KJ, Hamilton JA, Sini RL, Manning DL, Nicholson RI, deFazio A, Watts CK, Musgrove EA and Sutherland RL (1993) Expression and amplification of cyclin genes in human breast cancer. *Oncogene* **8**: 2127–2133

Cong J, Ardelt B, Traganos F and Darzynkiewicz Z (1994) Unscheduled expression of cyclin B1 and cyclin E in several leukemic and solid tumor cell lines. *Cancer Res* **54**: 4285–4288

Cordon-Cardo C (1995) Mutation of cell cycle regulators. Biological and clinical implications for human neoplasia. *Am J Pathol* **147**: 545–560

Ewen ME, Sluss HK, Sherr CJ, Matsushime H, Kato JY and Livingston DM (1993) Functional interaction of the retinoblastoma protein with mammalian D-type cyclins. *Cell* **73**: 487–497

Fahn H-J, Wang L-S, Huang B-S, Huang M-H and Chien K-Y (1994) Tumor recurrence in long-term survivors after treatment of carcinoma of the esophagus. *Ann Thorac Surg* **57**: 677–681

Furihata M, Ohtsuki Y, Ogoshi S, Takahashi A, Tamiya T and Ogata T (1993) Prognostic significance of human papillomavirus genomes (type-16, -18) and aberrant expression of p53 protein in human esophageal cancer. *Int J Cancer* **54**: 226–230

Furihata M, Ishikawa T, Inoue A, Yoshikawa C, Sonobe H, Ohtsuki Y, Araki K and Ogoshi S (1996) Determination of the prognostic significance of unscheduled cyclin A overexpression in patients with esophageal squamous cell carcinoma. *Clin Cancer Res* **2**: 1781–1785

Geradts J, Hu SX, Lincoln CE, Benedict WF and Xu H-J (1994) Aberrant RB gene expression in routinely processed, archival tumor tissues determined by three different anti-Rb antibodies. *Int J Cancer* **58**: 161–167

Gillett C, Fantl V, Smith R, Fisher C Bartek J, Dickson C, Barnes D and Peters G (1994) Amplification and overexpression of cyclin D1 in breast cancer detected by immunohistochemical staining. *Cancer Res* **54**: 1812–1817

Hermanek P, Sobin LH (eds) (1992) *UICC TNM classification of malignant tumors*, 4th edn, 2nd revision. Springer Verlag: Berlin

Hunter T and Pines J (1991) Cyclins and cancer. A review. *Cell* **66**: 1071–1074

Jares P, Fernandez PL, Campo E, Nadal A, Bosch F, Aiza G, Nayach I, Traserra J and Cardesa A (1994) *PRAD-1/Cyclin D1* gene amplification correlates with messenger RNA overexpression and tumor progression in human laryngeal carcinomas. *Cancer Res* **54**: 4813–4817

Jiang W, Zhang Y-J, Kahn SM, Hollstein MC, Santella RM, Lu SH, Harris CC, Montesano R and Weinstein IB (1993) Altered expression of the cyclin D1 and retinoblastoma genes in human esophageal cancer. *Proc Natl Acad Sci USA* **90**: 9026–9030.

Kato H, Tachimori Y, Watanabe H, Iizuka T, Terui S, Itabashi M and Hirota T (1991) Lymph node metastasis in thoracic esophageal carcinoma. *J Surg Oncol* **48**: 106–111

© Cancer Research Campaign 1998

Kato H, Tachimori Y, Watanabe H and Iizuka T (1993) Evaluation of the new (1987) TNM classification for thoracic esophageal tumors. *Int J Cancer* **53**: 220–223

Keyomarsi K, O'Leary N, Molnar G, Lees E, Fingert H J and Pardee AB (1994) Cyclin E, a potential prognostic marker for breast cancer. *Cancer Res* **54**: 380–385

Kitagawa Y, Ueda M, Ando N, Shinozawa Y, Shimizu N and Abe O (1991) Significance of *int 2/hst-1* coamplification as a prognostic factor in patients with esophageal squamous carcinoma. *Cancer Res* **51**: 1504–1508

Lipponen PK, Liukkonen TJO (1995) Reduced expression of retinoblastoma (Rb) gene protein is related to cell proliferation and prognosis in transitional-cell bladder cancer. *J Cancer Res Clin Oncol* **121**: 44–50

Matsushime H, Quelle DE, Shurtleff SA, Shibuya M, Sherr CJ and Kato JY (1994) D type cyclin-dependent kinase activity in mammalian cells. *Mol Cell Biol* **14**: 2066–2076

McIntosh GG, Anderson JJ, Milton I, Steward M, Parr AH, Thomas MD, Henry JA, Angus B, Lennard TWJ and Horne CHW (1995) Determination of the prognostic value of cyclin D1 overexpression in breast cancer. *Oncogene* **11**: 885–891

Michalides R, van Veelen N, Hart A, Loftus B, Wienjens E and Balm A (1995) Overexpression of cyclin D1 correlates with recurrence in a group of forty-seven operable squamous cell carcinomas of the head and neck. *Cancer Res* **55**: 975–978

Motokura T, Bloom T, Kim HG, Juppner H, Ruderman JV, Kronenberg HM and Arnold A (1991) A novel cyclin encoded by *BCL1*-linked candidate oncogene. *Nature* **350**: 512–515

Müller H, Lukas J, Schneider A, Warthoe P, Bartek J, Eilers M and Strauss M (1994) Cyclin D1 expression is regulated by the retinoblastoma protein. *Proc Natl Acad Sci USA* **91**: 2945–2949

Naitoh H, Shibata J, Kawaguchi A, Kodama M and Hattori T (1995) Overexpression and localization of cyclin D1 mRNA and antigen in esophageal cancer. *Am J Pathol* **146**: 1161–1169

Pagano M, Pepperkok R, Verde F, Ansorge W and Draetta G (1992) Cyclin A is required at two points in the human cell cycle. *EMBO J* **11**: 961–971

Pines J and Hunter T (1994) Cyclins and cancer II: cyclin D1 and CDK inhibitors come of age. *Cell* **79**: 573–582

Sherr CJ (1993) Mammalian G1 cyclins. *Cell* **73**: 1059–1065

Shew JY, Ling N, Yang X, Fodstad O and Lee WH (1989) Antibodies detecting abnormalities of the retinoblastoma susceptibility gene product ($pp^{110RB}$) in osteosarcomas and synovial sarcomas. *Oncogene Res* **1**: 205–214

Shimaya K, Shiozaki H, Inoue M, Thahara H, Monden T, Shimano T and Mori T (1993) Significance of p53 expression as a prognostic factor in oesophageal squamous cell carcinoma. *Virchows Arch A Pathol Anat Histol* **422**: 271–276

Shinozaki H, Ozawa S, Ando N, Tsuruta H, Terada M, Ueda M and Kitajima M (1996) *Cyclin D1* amplification as a new predictive classification for squamous cell carcinoma of the esophagus, adding gene information. *Clin Cancer Res* **2**: 1155–1161

Trudel M, Mulligan L, Cavenee W, Margolese R, Cote J and Gariepy G (1992) Retinoblastoma and p53 gene product expression in breast carcinoma: immunohistochemical analysis and clinicopathologic correlation. *Hum Pathol* **23**: 1388–1394

Tsuruta H, Sakamoto H, Onda M and Terada M (1993) Amplification and overexpression of *EXP1* and *EXP2/cyclin D1* genes in human esophageal carcinomas. *Biochem Biophys Res Commun* **196**: 1529–1536

Weinberg RT (1991) Tumor suppressor genes. *Science* **254**: 1138–1146

Withers D, Harvey R, Faust J, Melnyk O, Carey K and Meeker T (1991) Characterization of a candidate *bcl-1* gene. *Mol Cell Biol* **11**: 4846–4853

Xu HJ, Hu SX and Benedict WF (1991) Lack of nuclear RB protein staining in G0/G1 cells: correlation to changes in total RB protein level. *Oncogene* **6**: 1139–1146

Xu HJ, Hu SX, Hashimoto T, Takahashi R and Benedict WF (1989) The retinoblastoma susceptibility gene product: a characteristic pattern in normal cells and abnormal expression in malignant cells. *Oncogene* **4**: 807–812

Zang YG, Jiang W, Chen CJ, Lee CS, Kahn SM, Santella RM and Weinstein IB (1993) Amplification and overexpression of cyclin D1 in human hepatocellular carcinoma. *Biochem Biophys Res Commun* **196**: 1010–1016

© Cancer Research Campaign 1998